# Integrating tumor microenvironment with cancer molecular classifications

**DOI:** 10.1186/s13073-015-0241-4

**Published:** 2015-11-14

**Authors:** Etienne Becht, Aurélien de Reyniès, Wolf H. Fridman

**Affiliations:** INSERM UMR_S 1138, Cancer, Immune Control and Escape, Cordeliers Research Centre, Paris, France; Université Paris Descartes, Paris, France; Université Pierre et Marie Curie, Paris, France; Programme Cartes d’Identité des Tumeurs, Ligue Nationale Contre le Cancer, Paris, France

## Abstract

The composition of the tumor microenvironment is associated with a patient's prognosis and can be therapeutically targeted. A link between the cellular composition and genomic features of the tumor and its response to immunotherapy is beginning to emerge. Analyzing the microenvironment of tumor molecular subgroups can be a useful approach to tailor immunotherapies.

## The importance of the immune microenvironment

Cancer cells grow within a microenvironment where they interact with stromal cells (such as fibroblasts and endothelial cells) and immune cells. These interactions are of prime relevance for the outcome of patients with cancer. Our understanding of how the adaptive immune system controls tumor growth and metastatic spread has vastly improved in the past decade. An early example of these studies in colorectal cancer (CRC) showed that high densities of memory and cytotoxic T cells are associated with favorable patient prognosis, a result that has since been extended to a large number of other cancers [1]. Other adaptive immune cells were reported to be implicated in this anti-tumor mechanism, notably type 1 T helper (T_h_1) lymphocytes, which activate cytotoxic T cells, and B cells, which can produce tumor-targeting antibodies [1]. Lymphocytes form aggregates surrounding the tumor, an observation that was first made in non-small cell lung cancer, and these aggregates can be organized in tertiary lymphoid structures that structurally resemble secondary lymphoid organs (lymph nodes) where systemic immune responses are mounted. These structures seem to locally foster T_h_1/CD8 immune responses by locally enabling antigen presentation by mature dendritic cells (mDCs) [2].

These findings have since been translated into the clinic, with agents that stimulate the activity of cytotoxic T cells, such as immune checkpoint inhibitors, yielding clinical responses in patients with advanced-stage cancer. Immune checkpoints mostly consist of T-cell-expressed receptors (such as CTLA4 or PD-1), whose binding to ligands (such as PD-L1) suppresses T-cell activity. Tumor cells can coopt this mechanism to evade immune destruction, either by expressing inhibitory ligands themselves or by recruiting myeloid cells or other immune subsets that express these ligands. Anti-checkpoint treatments, such as antibodies that block these receptors or ligands, interfere with immunosuppressive signals to restore the anti-tumor potential of cytotoxic cells. These treatments elicit up to 30 % of objective responses in metastatic cancers [3], with the response rate depending on tumor type. It is of paramount importance to develop tumor classification systems able to predict responders to these treatments.

Pro-tumorigenic inflammation, another immune-mediated effect, has also been documented [4]. Inflammation signals mobilize the immune system in response to perturbations of tissue homeostasis, such as wounding or infection. Tumors can subvert inflammatory signals to sustain carcinogenesis by the production of mutagens, growth signals and angiogenic molecules, or by activation of matrix remodeling pathways [4]. Inflammation seems to have a role in the suppression of adaptive anti-tumor immune responses through stimulation of the production of regulatory T cells and suppressive myeloid cells, as well as the production of soluble immunosuppressive factors such as TGFß. Future successful immunotherapeutic approaches will be likely to aim at simultaneously restoring the adaptive immune response while decreasing the pro-tumorigenic inflammation. Understanding the immune microenvironment of tumors is therefore important in the development of tailored immunotherapies.

## Integrating immune and molecular classification of tumors

Prediction of response to immunotherapies has been a major goal in studies of the immune microenvironment. Our group characterized the immune microenvironment of pulmonary metastases from CRC and clear-cell renal cell carcinoma (ccRCC) [5]. This analysis revealed that, within the same surrounding pulmonary tissue, the immune cell densities found in the tumor microenvironment, as well as their associated prognostic values, are influenced by the metastasis-forming malignant cell(s). This finding suggests a critical role of these metastasis-forming malignant cells in shaping the tumor’s immune microenvironment. Therefore, we would expect to see a correlation between the molecular signature of a tumor cell and its immunological features.

Several types of cancer have now been divided into molecularly homogeneous subgroups, which are usually established using unsupervised classification of 'omics' data. These molecular signatures are often associated with genomic features of the tumors and clinical characteristics of the patients. To analyze the relationship between the immune microenvironment and molecular subgroups of various cancers, we developed a method to identify and measure the expression of genes specific to the main immune and stromal cell populations.

This method was first applied in a cohort of primary tumors from metastatic ccRCC, in which four molecular subgroups were identified [6]. This analysis revealed a significant association between ccRCC molecular subgroups and immune infiltrates. In particular, it revealed that a sunitinib-resistant subgroup with significantly shorter overall survival is highly infiltrated by cytotoxic T cells and expresses genes related to a T_h_1 functional orientation, as well as being highly infiltrated by cells of monocytic origin (macrophages) and expressing high levels of inflammatory, immunosuppressive and checkpoint molecules (PD-1 and its ligands and LAG3) [6, 7]. These observations indicate the presence of a highly inflammatory microenvironment in which anti-tumor effector cells are present but their activity is suppressed. The presence of effector cells in conjunction with the expression of checkpoint molecules suggests that the ccrcc4 molecular subgroup could be enriched for responders to inhibitors of the PD-1 pathway.

Many independent teams have proposed molecular classifications of CRC in the past few years. They all agree on the identification of a microsatellite-instable (MSI)-enriched subgroup associated with favorable prognosis, and a mesenchymal subgroup associated with poor prognosis [8]. Analysis of the immune microenvironment of molecularly classified CRC tumors strikingly revealed that these two subgroups with opposed prognosis are both highly infiltrated by immune cells [7]. The previously described immunological subgroup of CRC that was marked by extensive infiltration by cytotoxic T cells, with high expression of genes encoding memory T-cell chemoattractants or cytokines promoting cytotoxic T-cell-mediated immunity, closely corresponded to the MSI-enriched subgroup, whose genome is notable for its high mutational burden due to defects in the DNA repair machinery. This subgroup also had the highest expression of checkpoint molecules, such as PD-L1 and PD-L2, among all CRC subgroups studied, which suggests it could respond to anti-PD-1 treatments [7]. Subsequent reports confirmed this hypothesis, as MSI enrichment seems to be tightly correlated to response to pembrolizumab, a PD-1-targeting monoclonal antibody [9]. Strikingly, another report showed that in non-small cell lung cancer, the overall mutational load of the tumors is associated with response to PD-1 blockade [10]. Therefore, antigenicity (the capacity to elicit an adaptive immune response), which is tightly associated with the presence of DNA-encoded non-synonymous mutations, as well as with a cytotoxic orientation of the microenvironment, could be a major determinant of response to checkpoint inhibitors.

Combination of immunotherapies or drugs targeting other features of the tumor microenvironment might, however, benefit other subgroups of patients. The analysis of CRC molecular subgroups also revealed a previously unidentified 'immune-high' subgroup of CRC [7]: the poor-prognosis mesenchymal subgroup indeed expressed intermediate levels of markers of the adaptive immune response and checkpoint molecules, in conjunction with a high degree of infiltration by macrophages, high expression of inflammatory genes, high degree of angiogenesis and fibroblast infiltration, and abundance of soluble immunosuppressive molecules such as TGFß [7]. This pattern suggests that, similarly to the situation in poor-prognosis ccRCC tumors, high inflammation hampers the activity of cytotoxic cells in mesenchymal CRC tumors, and thus anti-inflammatory or anti-angiogenic treatments could be used in combination with checkpoint inhibitors to simultaneously dampen inflammatory signals and restore cytotoxic T-cell function in this subgroup.

Altogether, these data, which will be extended to other cancers, illustrate that molecular genome-wide and immune classifications of tumors are highly correlated, and that together they enable the discovery of different immune microenvironments within a given cancer that can be therapeutically targeted.

## Abbreviations

ccRCC: clear-cell renal cell carcinoma
CRC: colorectal cancer
mDC: mature dendritic cell
MSI: microsatellite instability
T_h_1: type 1 T helper
